# Enhanced Cycling Stability in Zn‐Ion Batteries Using Aqueous‐Organic Electrolyte Solvent Blends

**DOI:** 10.1002/advs.202507332

**Published:** 2025-07-17

**Authors:** Wei Huang, Ze He, Boya Cao, Minfei Fei, Xinjuan Li, Shaoliang Guan, Qing Dai, Rui Wang, Shijie Zhu, Xunyao Luo, Tianhao Wu, Simon Fairclough, Caterina Ducati, Michael De Volder

**Affiliations:** ^1^ Department of Engineering University of Cambridge Cambridge CB3 0FS UK; ^2^ Department of Materials Science and Metallurgy University of Cambridge Cambridge CB3 0FS UK; ^3^ Cavendish Laboratory University of Cambridge Cambridge CB3 0HE UK

**Keywords:** hybrid electrolytes, solid electrolyte interphase, zinc‐ion batteries

## Abstract

Zinc‐ion batteries (ZIBs) have emerged as a promising candidate for safe and affordable energy storage. This is particularly true for ZIBs using aqueous electrolytes, but unfortunately, they suffer from severe degradation issues that have not been resolved despite of extensive research efforts in this field. Hybrid electrolytes using both water and dimethyl sulfoxide (DMSO) as a solvent are proposed, which combine the non‐flammable properties of water‐based electrolytes with the stability of solvent‐based electrolytes. Further, this hybrid electrolyte allows for using tetrafluoroborate (Zn(BF_4_)_2_) as a salt, which readily corrodes Zn in aqueous formulations, but also contributes to a more stable SEI in the hybrid solvent system. These results are corroborated using Density Functional Theory (DFT) simulations to show how DMSO modifies the solvation structure, as well as HRTEM to analyze the SEI structure and composition. Finally, it is demonstrated that the hybrid electrolyte suppresses the dissolution of NVO cathodes, allowing Zn||NVO full cells to operate stably for over 2000 cycles with ≈80% capacity retention. Overall, this work illustrates how mixed solvent‐water based electrolytes have the potential to retain the safety and cost advantages of aqueous electrolytes, while at the same time suppressing degradation mechanisms.

## Graphical abstract

This paper combines Zn(BF4)₂ salt with a water‐DMSO solvent system to create a thin and stable SEI layer in Zinc‐Ion batteries, offering significant improvements in cycling stability over traditional aqueous electrolytes.

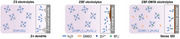

## 1 Introduction

Zn‐ion batteries (ZIBs) are a promising alternative to Lithium‐ion batteries (LIBs) in applications where weight and volume efficiency are of secondary importance such as in renewable energy storage. Compared to LIBs, ZIBs offer enhanced safety, primarily due to the use of non‐flammable aqueous electrolytes as well as better cost efficiency.^[^ ^1^, ^2^, ^3^ ^]^ Additionally, ZIBs do not put additional pressure on Li, Ni, and Co mining, which is critical to satisfy the need for EV battery production.^[^ ^4^, ^5^, ^6^ ^]^ However, ZIB's aqueous electrolytes are both their main strength and weakness as they are unstable at the Zn anode operating voltage (−0.763 V vs SHE), leading to hydrogen evolution reactions (HER).^[^ ^7^ ^]^ In particular, HER is accentuated by the presence of “active water” [Zn(H_2_O)_6_]^2+^,^[^ ^8^, ^9^ ^]^ which is reported to decompose into H^+^ and OH^−^ more readily, and H^+^ ions are likely to be reduced during Zn plating.^[^ ^10^, ^11^ ^]^ These issues have a significant impact on the performance and durability of ZIBs,^[^ ^12^, ^13^ ^]^ and therefore ZIB electrolytes require further investigation.

One solution to suppress HER is to use organic electrolytes,^[^ ^1^, ^14^, ^15^, ^16^ ^]^ in conjunction with salts such as Zn(TFSI)_2_ and Zn(CF_3_SO_3_)_2_. While impressive advances in ZIB cycling stability are achieved with these advanced electrolytes, they negate the safety and cost‐efficiency benefits that make ZIBs attractive in the first place. Zinc tetrafluoroborate (Zn(BF_4_)_2_), typically used as a flame retardant, emerged as a promising solute candidate for ZIBs. As shown in Figure S1 and Table S1 (Supporting Information), Zn(BF_4_)_2_ is a cost‐effective salt, but recent studies on organic solvent – Zn(BF_4_)_2_ electrolytes show limited cycling stability compared that of Zn(TFSI)_2_ and Zn(CF_3_SO_3_)_2_. Further, it was found that Zn(BF_4_)_2_ in aqueous electrolytes leads to low electrolyte pH values, leading to corrosion challenges.

**Figure 1:**
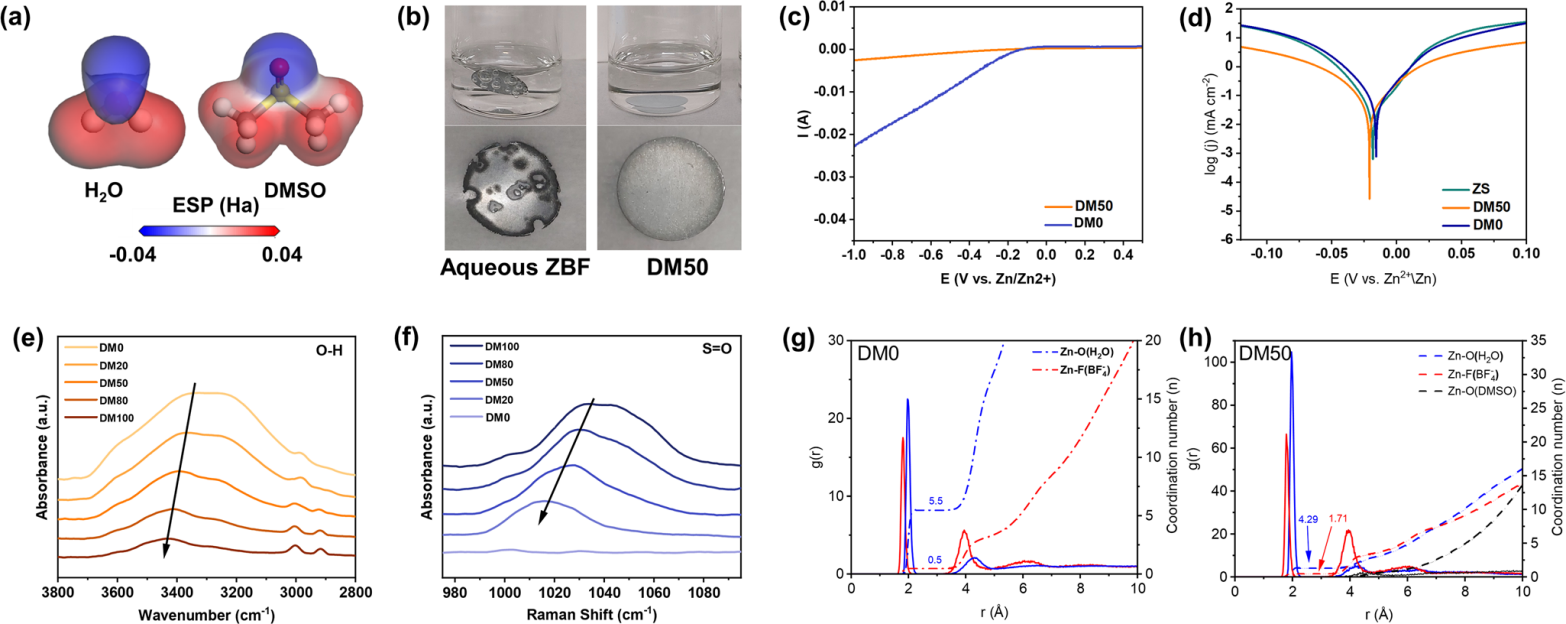
a) Electrostatic potential (ESP) maps of water and DMSO molecules. b) Zn foils after soaking in DM0 and DM50 for 2 days. c) Linear sweep voltammetry (LSV) of DM50 and DM0 at a scan rate of 5 mV s^−1^. d) Tafel plots derived from LSV test for DM0, DM50, and ZS. e) Fourier Transform Infrared Spectroscopy (FTIR) for DM0, DM20, DM50, DM80, and DM100. f) Raman spectroscopy for DM0, DM20, DM50, DM80, and DM100. g) CN and RDF results of DM0. h) CN and RDF results of DM50.

In this work, we propose to use a hybrid electrolyte using a mixture of water and DMSO to balance the benefits and drawbacks of aqueous and organic systems. DMSO is a cost‐effective organic solvent often used as a cellular preservative and freezing medium due to its complete miscibility with water and its ability to significantly lower the melting point.^[^ ^17^, ^18^ ^]^ Owing to its high polarity (**Figure** **1**a), it also changes the solvation structure of the salt and as a result, reduces water molecule activity in the electrolyte and HER.^[^ ^18^ ^]^ We screen DMSO‐H_2_O electrolyte mixtures with a DMSO molar ratio of 0%, 20%, 50%, 80%, and 100% (denoted as DM0, DM20, DM50, DM80, DM100) and focus on Zn(BF_4_)_2_ salt (0.5 M). Further, we show that these hybrid co‐solvent systems modify the solvation structure using DFT simulations and result in a different SEI composition using HRTEM. Symmetric cells with this electrolyte achieved an exceptional cycling stability of 3000 h under practical cycling conditions (3 mA cm^−2^ and 3 mAh cm^−2^). Additionally, Zn||NVO full cells also achieve a capacity retention of 80% for 2000 cycles.

## 2 Results and Discussion

To assess the corrosion resistance of Zn anodes in the proposed hybrid electrolyte, a soaking experiment was conducted. As shown in Figure 1b, Zn anodes immersed in the DM0 exhibited significant corrosion, with holes and bubbles formed across the 38 µm‐thick Zn disks, as well as black deposits forming on the edge of the electrodes. Conversely, Zn anode immersed in the hybrid electrolyte (DM50) retained its intact morphology, suggesting a markedly improved corrosion resistance. Notably, this intact surface morphology was also well maintained after long‐term cycling in DM50, further confirming the excellent interfacial stability of the hybrid electrolyte system (Figure S2, Supporting Information). One possible explanation for the observed corrosion in DM0 is the hydrolysis of the tetrafluoroborate anion (BF_4_^−^), which could produce hydrofluoric acid (HF), a highly corrosive substance capable of attacking zinc.^[^ ^19^ ^]^ This reaction may lead to the formation of ZnF_2_ and H_2_, driving the severe corrosion observed in DM0.^[^ ^20^ ^]^ Our pH measurements provide supporting evidence for this hypothesis, revealing that DM0 had a pH of 0.88, suggesting significant BF_4_^−^ hydrolysis and HF generation could happen (Figure S3, Supporting Information). In contrast, DM50 exhibited a much higher pH of 4.3, indicating that the addition of DMSO may suppress the hydrolysis of BF_4_^−^ and the associated HF formation, thereby mitigating the corrosion reactions described above.

To further investigate corrosion and the HER in these electrolytes, linear sweep voltammetry (LSV) analyses were performed. As shown in Figure 1c, the hybrid electrolyte (DM50) exhibits a largely suppressed HER. The onset of the HER in the DM0 is −0.052 (V vs Zn/Zn^2+^ at −0.01 A), while the leakage current density was still negligible even at −0.6 (V vs Zn/Zn^2+^) for DM50. Figure 1d shows Tafel plots for a ZnSO_4_/H_2_O reference electrolyte (denoted as ZS) as well as DM0 and DM50. DM0 yields a corrosion current density of 1.0 mA cm^−2^ and ZS an even higher corrosion current density of 4.0 mA cm^−2^. In contrast, DM50 hybrid electrolytes markedly suppressed the corrosion kinetics, as evidenced by a more positive corrosion potential and a tenfold reduction in corrosion current density compared to ZS (0.41 mA cm^−2^).

The electrostatic potential (ESP) map of DMSO was calculated through DFT, where a strong localization of electron density around the ─S═O group was observed (Figure 1a).^[^ ^21^ ^]^ This concentration of negative charge on the sulphur atom not only facilitates the attraction toward positively charged sites on the molecule but also may act as a favorable site for hydrogen bonding with water molecules. To confirm that DMSO reduces the presence of active water, we studied FTIR and Raman signatures for active water in electrolytes with different water‐to‐DMSO ratios. In the FTIR spectrum of aqueous electrolyte, a broad peak ranging from 3000 to 3600 cm^−1^ was observed, attributable to the O─H stretching vibrations in water molecules.^[^ ^22^ ^]^ After adding increasing ratios of DMSO to the electrolyte, a shift of the O─H peak to higher wavenumbers is noted, indicating an enhancement in the strength of O─H bonding and a concomitant weakening of the hydrogen bonding network within the water molecules (Figure 1e).^[^ ^23^ ^]^ This observation is consistent with the intermolecular interaction dynamics, where DMSO acts as a hydrogen bond acceptor, thereby altering the hydrogen bond structure of water.^[^ ^17^ ^]^ The second derivative of the FTIR spectra reinforces this observation, revealing distinct vibrational modes corresponding to network water, intermediate water, and free water, all of which exhibit blue‐shifted features upon DMSO addition (Figure S4, Supporting Information). This shift indicates attenuated hydrogen bonding, as DMSO molecules disrupt the hydrogen‐bonding network by preferentially coordinating with water, thereby weakening the water–water interactions.^[^ ^23^, ^24^ ^]^ Complementing this, the Raman spectra revealed a downward shift in the S═O bonds of DMSO, which further suggests the possible formation of the new S═O….H─O hydrogen bonds (Figure 1f).^[^ ^17^, ^25^ ^]^

The solvation structure of Zn^2+^ was further investigated by molecular dynamics (MD) simulations. As shown in Figure S6 (Supporting Information), in the baseline ZnSO_4_/H_2_O electrolyte, the coordination number (CN) between Zn^2+^ and H_2_O is ≈5.9, with a radial distribution function of ≈2 Å, which agrees with other studies.^[^ ^26^, ^27^ ^]^ This aligns well with the classical [Zn(H_2_O)_6_]^2+^ structure. A similar solvation structure was observed in the electrolyte with Zn(BF_4_)_2_ (Figure S5, Supporting Information). In the Zn(BF_4_)_2_/H_2_O electrolyte, an initial CN of 5.5 was obtained between Zn^2+^ and H_2_O. This indicates a significant presence of active water molecules in the aqueous solvation shell (Figure 1g). In contrast, within the DM50 electrolyte, the CN between Zn^2+^ and H_2_O was reduced to 4.3, with a CN of 1.7 between Zn^2+^ and BF_4_^−^, forming a [Zn(BF_4_^−^)_2_(H_2_O)_4_] complex (Figure 1h). This MD result confirms that DMSO molecules are largely excluded from the first solvation shell of Zn^2+^. Consequently, the solvation environment evolves toward more strongly associated species. Further analysis of the ion‐pairing structures based on molecular dynamics trajectories reveals that the proportion of anion aggregates (AGGs) increases from 5% in DM0 to 20% in DM50 (Figure S7, Supporting Information), accompanied by a moderate decline in SSIP and CIP populations. This increase in ion association is expected to slightly reduce the mobility and desolvation kinetics of Zn^2+^ during charge and discharge, as AGG‐type structures typically exhibit lower transport efficiency and higher desolvation energy.^[^ ^28^ ^]^ This reduced desolvation kinetics may contribute to a more uniform SEI formation and improved interfacial stability. The electrolyte therefore becomes less prone to deleterious side reactions.

**Figure 2:**
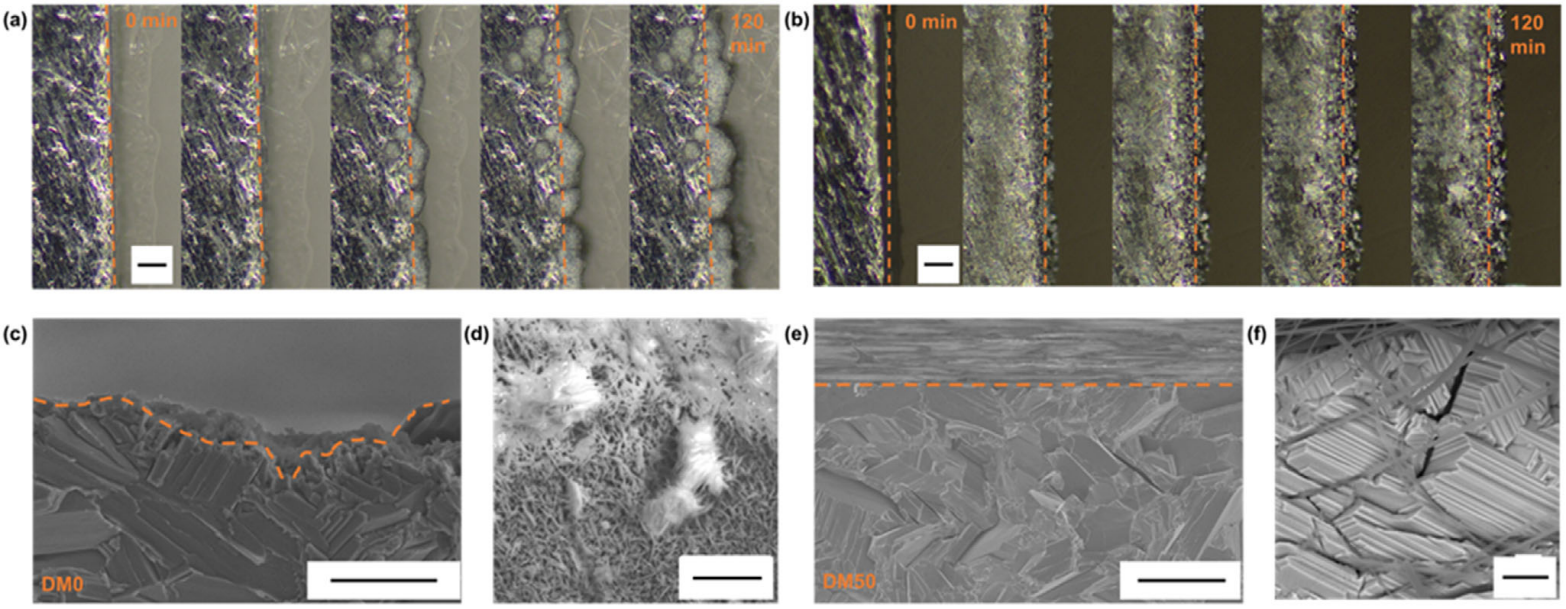
In situ optical microscopy images of Zn anodes tested at 3 mA cm^−2^ and 6 mAh cm^−2^ for: a) 0.5 m Zn(BF_4_)_2_ in H_2_O (DM0), after 1 plating cycle (scale bar: 50 µm); b) 0.5 M Zn(BF_4_)_2_ in H_2_O/DMSO (DM50), after 1 plating cycle (scale bar: 50 µm). c,d) SEM images for Zn anode after 5 cycles at 3 mA cm^−2^ and 3 mAh cm^−2^ in 0.5 m Zn(BF_4_)_2_ in H_2_O (DM0): (c) cross‐sectional image with scale bar of 20 µm; (d) top‐view image with scale bar of 2 µm. e,f) SEM images for Zn anode after 5 cycles at 3 mA cm^−2^ and 3 mAh cm^−2^ in DM50: (e) cross‐sectional image with scale bar of 20 µm; (f) top‐view image with scale bar of 2 µm.

To investigate the surface morphology evolution during the stripping/plating process, in situ optical microscopy was employed. An optical cell was subjected to a current density of 3 mA cm^−2^. When the Zn anode was cycled in DM0, uneven Zn deposition and extensive growth of protrusions were observed during the process (**Figure** **2**a). In contrast, Zn deposition in the hybrid electrolyte (DM50) seems less porous with significantly reduced surface roughness (Figure 2b). Furthermore, SEM micrographs were taken of the Zn surface after 5 cycles at 3 mA cm^−2^. These Zn anodes were cycled in the symmetric cells and then rinsed with deionized water. Cross‐sectional imaging of the Zn anode cycled in aqueous Zn(BF_4_)_2_ revealed a rough surface characterized by large pits (Figure 2c). Top‐view imaging (Figure S8, Supporting Information) further showed that these by‐products and corrosion pits were distributed across the entire surface. At higher magnification (Figure 2d), needle‐like crystals are observed in the DM0 electrodes, which are probably ZnF_2_ precipitates (see below) formed as a side product of the hydrolysis and HF etching discussed above. In contrast, cross‐sectional imaging of the Zn anode cycled in the DM50 electrolyte shows a flatter and smoother surface morphology after cycling (Figure 2e). Additionally, the plated Zn appeared more uniform (Figure 2f).

**Figure 3:**
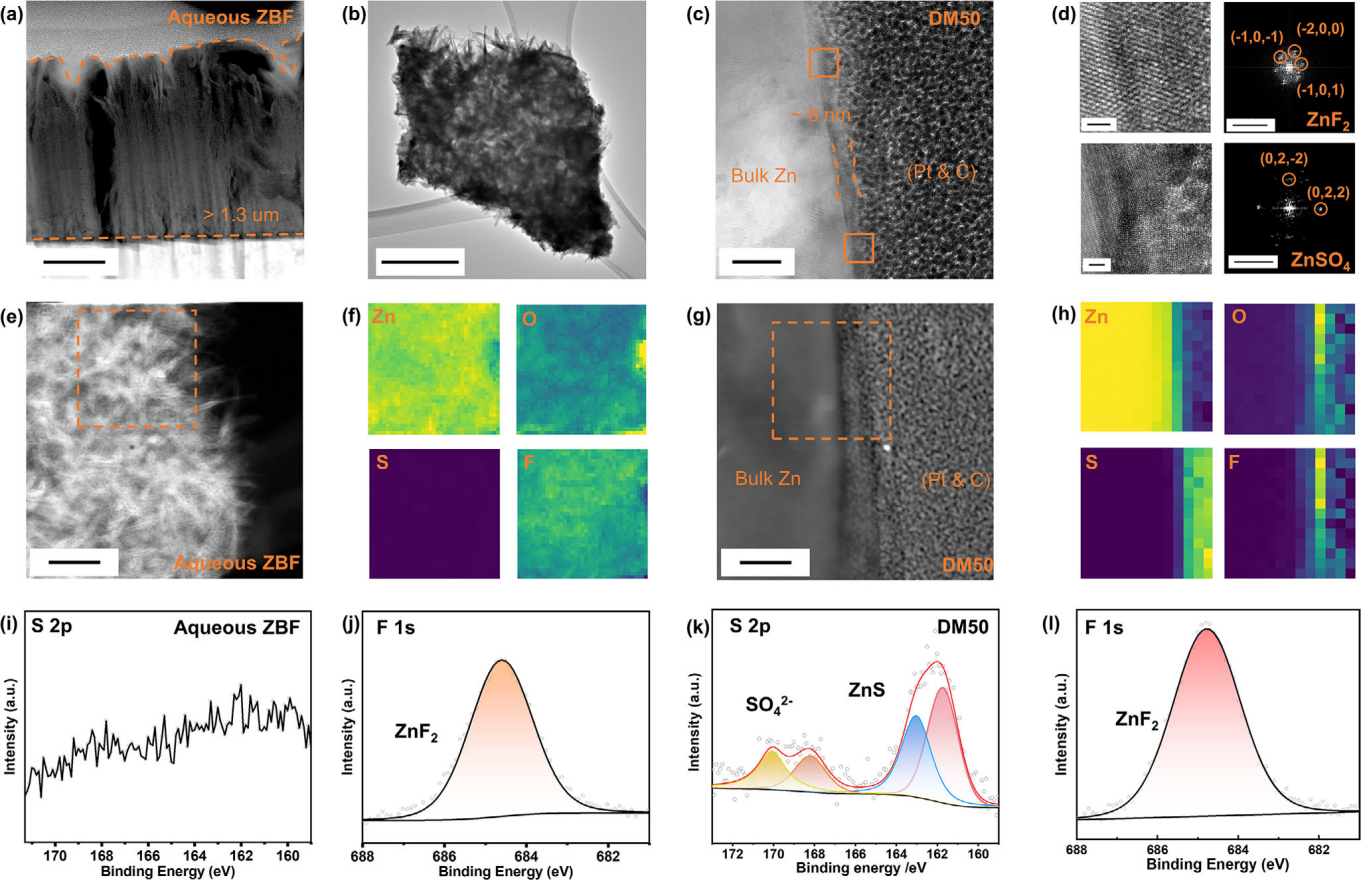
a) High‐angle annular dark‐field (HAADF) imaging of Zn anode cycled in DM0 for 5 cycles at 1 mA cm^−2^ and 1 mAh cm^−2^ (scale bar: 500 nm), lifted out by focused ion beam (FIB). b) HR‐TEM image of side products on the Zn anode after cycling in the DM0 for 5 cycles at 1 mA cm^−2^ and 1 mAh cm^−2^ (scale bar: 500 nm). c) HR‐TEM image of Zn anode after cycling in the DM50 electrolyte for 5 cycles at 1 mA cm^−2^ and 1 mAh cm^−2^ (scale bar: 20 nm & 10 1/nm). d) Diffraction patterns derived from different regions of the thin SEI layer in Figure 3c (scale bar: 1 nm) using Fast Fourier Transform (FFT). e,f) HAADF image and the corresponding energy dispersive X‐ray spectroscopy (EDX) maps for elemental distribution of the Zn anode cycled in DM0 (scale bar: 200 nm). g,h) HAADF image and the corresponding energy dispersive X‐ray spectroscopy (EDX) maps for elemental distribution of the Zn anode cycled in DM50 at 1 mA cm^−2^ and 1 mAh cm^−2^. i,j) XPS results of S 2p and F 1s for Zn anode cycled in DM0. k,l) XPS results of S 2p and F 1s for Zn anode cycled in DM50.

Transmission electron microscopy (TEM) was employed to examine the elemental composition and structure of the anode's surface area. The Zn electrodes were cycled at 1 mA cm^−2^ and 1 mAh cm^−2^ for 5 cycles in either DM0 or DM50, then rinsed with deionized water multiple times to remove any residual electrolyte or salts. Cross‐sections of the corroded areas were lifted out using Focused Ion Beam (FIB). As shown in **Figure** **3**a, the high‐angle annular dark‐field (HAADF) image reveals that after 5 cycles in DM0, a non‐uniform corrosion layer ≈1.3 µm thick formed on the bulk Zn metal surface, which seemingly contains vertical channels or cracks. To facilitate detailed TEM analysis, a small portion of this corrosion layer was carefully removed from the anode surface using a blade tool. A fragment of the corrosion layer was successfully transferred to a TEM grid (Figure 3b), where needle‐like crystals, consistent with those observed in previous SEM images, were identified. The HAADF image and corresponding Energy dispersive X‐ray (EDX) maps (Figure 3e,f) indicate that this surface layer cycled in DM0 primarily consists of Zn, O, and F, with negligible S signal. In contrast, a much thinner SEI layer, ≈8 nm thick, was observed in the cross‐section lifted out from the anode cycled in the hybrid electrolyte (DM50) (Figure 3c). Fast Fourier Transform (FFT) analysis of the near‐surface region revealed diffraction patterns characteristic of ZnF_2_ and ZnSO_4_ within the thin SEI layer (Figure 3d). Notably, while ZnF_2_ forms due to the hydrolysis of BF_4_^−^, the presence of ZnSO_4_ can be attributed to the reaction of Zn^2+^ with sulfur species generated from the decomposition of DMSO, as there was no ZnSO_4_ initially present in the electrolyte.^[^ ^29^ ^]^ The corresponding HAADF and EDX images (Figure 3g,h) also confirmed the presence of F and S signals in the SEI.

Furthermore, X‐ray photoelectron spectroscopy (XPS) analysis was conducted to investigate the surface of the cycled Zn anodes (Figure S9, Supporting Information). For the anode cycled in DM0, F 1s signals corresponding to ZnF_2_ were observed at 684.6 eV (Figure 3j).^[^ ^30^ ^]^ Similarly, the anode cycled in the hybrid electrolyte (DM50) also exhibited a significantly F 1s signal indicating a ZnF_2_ presence (Figure 3l). Moreover, the S 2p spectrum (Figure 3k) confirmed the presence of ZnSO_4_ (168.9 eV) in the solid electrolyte interphase (SEI) and revealed a substantial amount of ZnS (162.2 eV), which both are a result of DMSO decomposition.^[^ ^18^, ^31^ ^]^ The XPS results indicate that while the addition of Zn(BF_4_)_2_ leads to ZnF_2_ formation in both aqueous and hybrid ZIBs, the characteristics of the SEI differ significantly. In DM0, the surface layer is non‐uniform, seemingly porous, and relatively thick, which aligns with literature, suggesting that BF_4_^−^ hydrolyses to form HF which reacts with Zn to form ZnF_2_ that precipitates locally. In contrast, the hybrid electrolyte system shows a thinner SEI (app. 10 nm) composed of ZnF_2_, ZnS, and ZnSO_4_. XPS depth profiling (Figure S10, Supporting Information) reveals that ZnSO_4_ is mainly distributed near the top surface SEI region, while ZnS and ZnF_2_ are more uniformly present throughout the depth. This result suggests that early‐stage interfacial reactions involving decomposition of electrolyte components may produce SO_4_^2−^ species that contribute to SEI formation. According to previous studies, the presence of multiple components can exhibit a synergistic effect that significantly enhances the stability of SEI layer.^[^ ^32^, ^33^, ^34^, ^35^ ^]^

**Figure 4:**
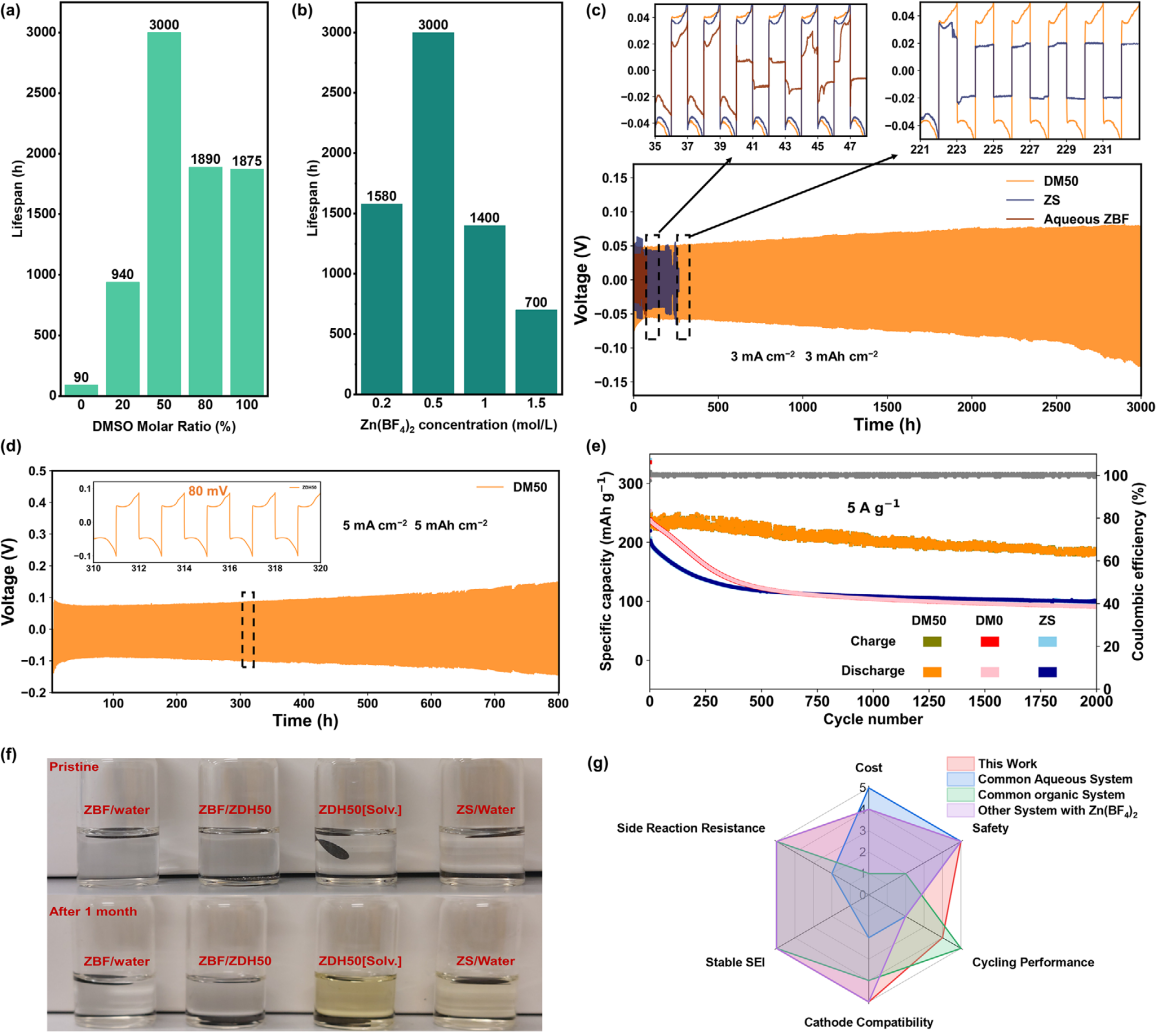
Cycling lifespan of symmetric Zn||Zn cells at 3 mA cm^−2^ current density with a 3 mAh cm^−2^ areal capacity as a function of a) DMSO molar ratio (%) and b) Zn(BF_4_)_2_ concentration (cycling data in Figure S15, Supporting Information). c) Zn||Zn symmetric cells cycled at 3 mA cm^−2^ and 3 mAh cm^−2^ for DM50, DM0, and ZS. d) Zn||Zn symmetric cells cycled at 5 mA cm^−2^ and 5 mAh cm^−2^. Electrochemical behavior of Zn||NVO full cells. e) Charge–discharge performance of the Zn||NVO cell with different electrolytes at a current density of 5 A g^−1^. f) Optical images of NVO cathodes immersed in different electrolytes, pristine (top) and after 1 month (bottom). g) Comparison of this work with other electrolyte systems based on cost, safety, cycling performance, cathode compatibility, stable SEI, and resistance to side reactions.

To optimize the electrolyte compositions, the salt concentration and solvent ratios are varied, and then cycled in symmetric cells, as shown in **Figure** **4**a,b. The results indicate that cycling stability is significantly affected by both the salt concentration and solvent ratio, where at a concentration of 0.5 m Zn(BF_4_)_2_ the DMSO:H_2_O molar ratio of 1:1 (DM50) shows the best performance. Next, the stability at a current density of 3 mA cm^−2^ and areal capacity of 3 mAh cm^−2^ was tested in symmetric cells. As shown in Figure 4c, the cells using DM0 showed a short lifespan of less than 40 cycles whereas the hybrid electrolyte (DM50) exhibits an ultra‐stable cycling lifespan of more than 1500 cycles (3000 h) with a low overpotential of 50 mV. In comparison, the most used aqueous ZnSO_4_ electrolyte (ZS) achieved a much shorter life of 110 cycles (220 h). It is worth mentioning that although DM50 exhibits a longer cycling life and a thinner SEI layer, its overpotential is slightly higher than that of DM0. This is because the intrinsic ionic conductivity of the hybrid electrolyte with added DMSO solvent is relatively lower compared to the aqueous electrolyte (Figure S11, Supporting Information). Therefore, achieving an appropriate DMSO ratio is particularly important. Additionally, even at the stressed cycling condition of 5 mA cm^−2^ and 5 mAh cm^−2^, a long cycling life of 400 cycles (800 h) was achieved with an overpotential of 80 mV (Figure 4d). Our hybrid electrolyte was also tested in Cu||Zn cells at 3 mA cm^−2^ and 3 mAh cm^−2^, yielding an average Coulombic Efficiency (CE) of 99.3% over 150 cycles (Figure S12, Supporting Information). To further evaluate the rate‐dependent and long‐term stability of the SEI, Zn||Zn symmetric cells were cycled at high current densities of 3 mA cm^−2^ (3C), 5 mA cm^−2^ (5C), and 10 mA cm^−2^ (10C) with a fixed capacity of 1 mAh cm^−2^. All systems maintained stable voltage profiles for over 200 h, indicating robust interfacial integrity across a wide range of cycling rates (Figure S13, Supporting Information). Notably, ex situ XPS analysis of Zn anodes after 10C cycling for 10 h and 1C cycling for 300 h revealed a similar SEI composition to that observed at earlier stages, with ZnF_2_, ZnS, and sulfate‐containing species as dominant components (Figure S14, Supporting Information), suggesting good structural and chemical consistency of the SEI under fast‐charging and long‐term cycling conditions.

Next, Zn||NaV_3_O_8_·1.5H_2_O (NVO) full cells were tested at 5 A g^−1^ (Figure 4e). The initial discharge capacity was 230 mAh g^−1^ after formation, and these full cells subsequently maintained ≈80% capacity retention over 2000 cycles. In contrast, the reference DM0 and ZnSO_4_/H_2_O (ZS) electrolytes exhibited significantly poorer cycling stability, with the DM0 system retaining only 38% and the ZS system 45% of their initial capacity after 2000 cycles (Charge–discharge curves shown in Figure S16, Supporting Information). Given these cycling results, we investigated if our proposed electrolyte also offers benefits to the battery cathode. More specifically, some electrolytes have been reported to dissolve NVO cathodes, which we tested by soaking our cathodes for one month in different electrolyte formulation (Figure 4f) and monitored changes in the color of the electrolyte as an indication of cathode dissolution. Specifically, the electrolytes using Zn(BF_4_)_2_ salt, including Zn(BF_4_)_2_/DMSO/H_2_O and Zn(BF_4_)_2_/H_2_O, remain transparent even after 30 days of immersion, whereas the electrolytes using ZnSO_4_ or DMSO/H_2_O solvent without Zn(BF_4_)_2_ exhibited a noticeable yellow discoloration. These observations indicate that Zn(BF_4_)_2_ not only ensures a stable anode but also exhibits excellent compatibility with vanadium‐based cathodes. Moreover, to compare this work with other electrolyte systems, we conducted a comparison of electrolytes across several key parameters (Figure 4g), against common aqueous systems, common organic systems, and other systems utilizing Zn(BF_4_)_2_ electrolytes. As shown in Tables S2 and S3 (Supporting Information), compared to other electrolyte systems, the DMSO/H₂O hybrid electrolyte introduced in this work offers a balanced solution that retains the low cost and safety advantages of Zn(BF_4_)_2_ while addressing the performance limitations of both aqueous and organic systems.^[^ ^36^, ^37^, ^38^, ^39^, ^40^, ^41^ ^]^

## 3 Conclusion

In summary, Zn(BF_4_)_2_ is a promising salt for ZIBs due to its low cost and fire‐retardant properties. However, in aqueous Zn(BF_4_)_2_ electrolytes, the anion hydrolyses, resulting in the formation of HF that etches Zn and leads to thick ZnF_2_ deposits. In this work, we introduced DMSO into the aqueous system, which suppresses Zn(BF_4_)_2_ hydrolysis and vice versa, this salt also acts as a fire retardant. Further, DMSO changes the solvation structure of Zn^2+^, which suppresses HER and facilitates the formation of a thin multi‐component SEI. Our optimized electrolyte operates for 3000 h at a practical test condition of 3 mA cm^−2^ and 3 mAh cm^−2^ in symmetric cells. Further, the inclusion of Zn(BF_4_)_2_ in the electrolyte also suppresses NVO dissolution, enabling a high capacity retention (80%) after 2000 cycles in Zn||NVO full cells.  

Supporting Information

The authors have cited additional references within the Supporting Information.^[^ ^20^, ^36^, ^37^, ^38^, ^39^, ^42^, ^43^, ^44^, ^45^, ^46^ ^]^

## Conflict of Interest

The authors declare no conflict of interest.

## Supporting information



Supporting Information

## Data Availability

The data that support the findings of this study are available from the corresponding author upon reasonable request.
