# Supplementary material for: Enhanced Cycling Stability in Zn‐Ion Batteries Using Aqueous‐Organic Electrolyte Solvent Blends
Source: Adv Sci (Weinh). 2025 Jul 17;12(38):e07332. doi: 10.1002/advs.202507332 (PMC12520558; doi:10.1002/advs.202507332)
Supplement: Supplementary file 1 — Supporting Information [file ADVS-12-e07332-s001.pdf]

ADVANCED SCIENCE
Open Access

Supporting Information

for *Adv. Sci.*, DOI 10.1002/advs.202507332

Enhanced Cycling Stability in Zn-Ion Batteries Using Aqueous-Organic Electrolyte Solvent Blends

Wei Huang, Ze He, Boya Cao, Minfei Fei, Xinjuan Li, Shaoliang Guan, Qing Dai, Rui Wang, Shijie Zhu, Xunyao Luo, Tianhao Wu, Simon Fairclough, Caterina Ducati* and Michael De Volder**

Supplementary Information

Cost-effective hybrid electrolyte design with a dense SEI layer enabling durable Zn-ion batteries

Wei Huang,[1,2] Ze He,[1]* Boya Cao,[1] Minfei Fei,[2] Xinjuan Li,[2] Shaoliang Guan,[2,3] Qing Dai,[1] Rui Wang,[1] Shijie Zhu,[3] Xunyao Luo,[3] Tianhao Wu,[2] Simon Fairclough,[2] Caterina Ducati,[2]* Michael De Volder,[1]*

[1]Institute for Manufacturing, Department of Engineering, University of Cambridge, Cambridge CB3 0FS, UK
[2]Department of Materials Science and Metallurgy, University of Cambridge, Cambridge CB3 0FS, UK
[3]Cavendish Laboratory, University of Cambridge, Cambridge CB3 0HE, UK

Experimental details

Materials & Battery Preparation

Initially, a solution composed of pure Dimethyl sulfoxide (DMSO, 99.9%) and water was prepared with different molar ratios, including 0%DMSO, 20%DMSO, 50%DMSO, 80%DMSO, and 100%DMSO. Subsequently, hydrated zinc tetrafluoroborate powders ($Zn(BF_4)_2 \cdot 7H_2O$) were dissolved in this DMSO-H_2O solvent at various concentrations (0.2 M, 0.5 M, 1 M, 1.3 M, 1.4 M) and stirred for 10 minutes, yielding the $Zn(BF_4)_2$-DMSO-H_2O electrolyte solutions. Additionally, as a reference electrolyte, zinc fluoride powders ($ZnSO_4$, 99%) were dissolved in deionized water to create a 1 M $ZnSO_4$ solution, referred to as ZS.

To prepare the battery anodes, zinc foil with a thickness of 38 nm was utilized and cut into small discs using a pressure gauge. On the other hand, to fabricate the battery cathodes, a slurry consisting of V_2O_5 powder, conductive carbon (super P), and Polyvinylidene Fluoride (PVDF, Solef 6020) was blended with a mass ratio of 6:3:1. The resulting cathode material had a confirmed composition of $NaV_3O_8 \cdot 1.5H_2O$ (referred to as NVO), which were subsequently applied onto

Toray carbon paper (TGP-H-60). A mass loading of 2 mg/cm^2 on the carbon paper was adopted according to the standard established by Yuan Shang et al. in 2023 [1]. After that, the prepared plate was dried in a vacuum at 60 °C for 24 hours and subsequently cut into discs to form the cathode.

Coin cells were then assembled, employing 2032 stainless steel coin cells, with glass fiber papers (GF/A) as separators, which thoroughly soaked in 72 uL of the electrolyte.

Electrochemical Measurements & Characterisation

Galvanostatic plating/stripping curves were generated using the LAND battery testing system (CT2001A). Two different types of plating/stripping process were conducted, namely Zn//Zn symmetric cycling and Zn//NVO full cell cycling. Zn//Zn symmetric cycling, involving alternate charging (plating) and discharging (stripping) of zinc foils acting as both electrodes, was employed as an initial assessment of the zinc anode's cycling stability in varied electrolytes. The stability was primarily determined by monitoring the overpotential during repeated cycles, as well as the overall lifespan of the cells. While the Zn//Zn symmetric cycling is instrumental in evaluating the cycling stability of zinc anodes in various electrolytes, it may not fully reflect the real-world application of the electrolyte within a complete battery system. Thus, the Zn//NVO full cell cycling was introduced as a parallel assessment to ensure a holistic evaluation that aligns with practical battery operations. This evaluation was facilitated by the use of a vanadium-based cathode (NVO) and measured through the residual capacity retained by the cathode after each cycle, which mirrors the compatibility of the vanadium cathode with the targeted electrolyte, providing more insights into the practical viability of the electrolyte in ZIBs. The Linear Sweep Voltammetry (LSV) measurements were also performed on this electrochemical workstation (BCS-805), using a scan rate of 5 mV/s and a voltage range of -0.2 V to 0.2 V.

The Fourier Transform Infrared Spectroscopy (FT-IR) was recorded at room temperature using a PerkinElmer sm2205 spectrometer. The Raman spectrum was tested on a renishaw inVia™ Raman microscope using 532 nm laser.

Optical microscopy images were captured using a BX53M (Olympus) microscope, which allows for both reflected and transmitted light imaging. Images were recorded with an LC30 camera, utilizing either darkfield or brightfield illumination modes.

The surface morphology of zinc anodes was characterized using a Scanning Electron Microscope (SEM, Phenom pro) at an acceleration voltage of 10 kV. Cross-sectional lamellae suitable for transmission electron microscopy (TEM) analysis were prepared via focused ion beam (FIB) milling. TEM data were collected using an FEI Tecnai Osiris TEM, which features a high-brightness Schottky X-FEG source and a Super-EDX system with four silicon drift detectors. Scanning/transmission electron microscopy (S/TEM) images were captured at an acceleration voltage of 200 kV.

XPS Analysis was performed using a Thermo NEXSA G2 XPS fitted with a monochromated Al kα X-ray source (1486.7 eV), a spherical sector analyser and 3 multichannel resistive plate, 128 channel delay line detectors. All data was recorded at 19.2W and an X-ray beam size of 400 x 200 µm. Survey scans were recorded at a pass energy of 200 eV, and high-resolution scans recorded at a pass energy of 50 eV. Electronic charge neutralization was achieved using an ion source (Thermo Scientific FG-03). Ion gun current = 150 µA. Ion gun voltage = 40 V. All sample data was recorded at a pressure below 10-8 Torr and a room temperature of 294 K. Data was analysed using CasaXPS v2.3.26rev1.0N. Peaks were fit with a Shirley background prior to component analysis. Lineshapes of LA (1.53,243) were used to fit components.

Simulation

For the Density Functional Theory (DFT) simulation, all calculations were

conducted using the Materials Studio (MS) in the Cambridge Sequential Total Energy Package (CASTEP) module. The generalized gradient approximation (GGA) functional by the Perdew-Burke-Ernzerhof (PBE) was employed. The cutoff energy was 489.8 eV, the pseudopotentials were OTFG ultrasoft, and the corresponding k-point sampling was 2×2×1 for geometry optimization. The Broyden–Fletcher–Goldfarb– Shanno (BFGS) scheme was selected as the minimization algorithm. The DFT-D method of Grimme was adopted to correct the van der Waals interaction. The convergence tolerance of energy was 2×10^{-5} eV per atom, the maximum force was 0.05 eV/Å, the maximum stress was 0.1 Gpa, the maximum displacement was 0.002 Å.

Molecular dynamics (MD) simulations were performed using the Materials Studio Forcite modules. Molecules and ions including H_2O, Zn^{2+}, BF_4^-, and DMSO were built and got geometry optimization through $Dmol^3$ software. Electrolyte systems were built by Amorphous Cell module. COMPASSIII force field was adopted for all molecules and ions. NPT ensemble was used to pre-equilibrate the systems at 298 K over a period of 1 ns, and then NVT ensemble was used to achieve a final equilibrate state of the systems at 298 K over a period of 5 ns. The final 1 ns in NVT production steps were sampled for radial distribution function (RDF) and coordination structure counting analyses.

Supplementary Figures

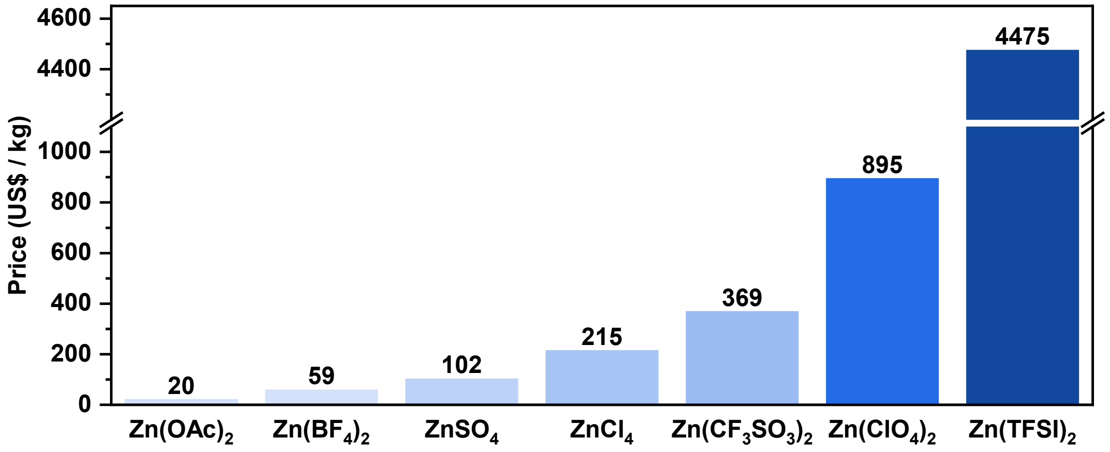

Figure S1. Estimated market price of different solutes used in ZIBs (all prices, with the same package size, were obtained from two major chemical suppliers in China, Aladdin and Aicaigou).

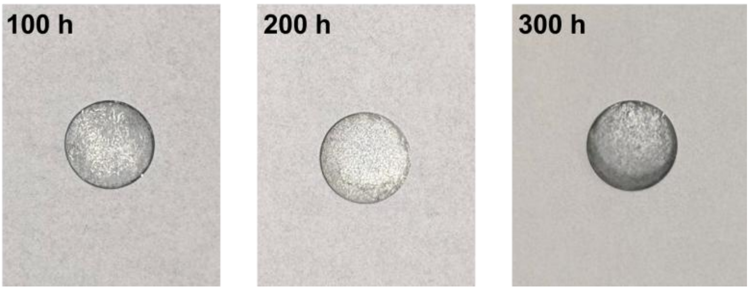

Figure S2. Zn electrode after cycling in DM50 for 100, 200, and 300 hours at 3 mA cm^{-2} 3 mAh cm^{-2}.

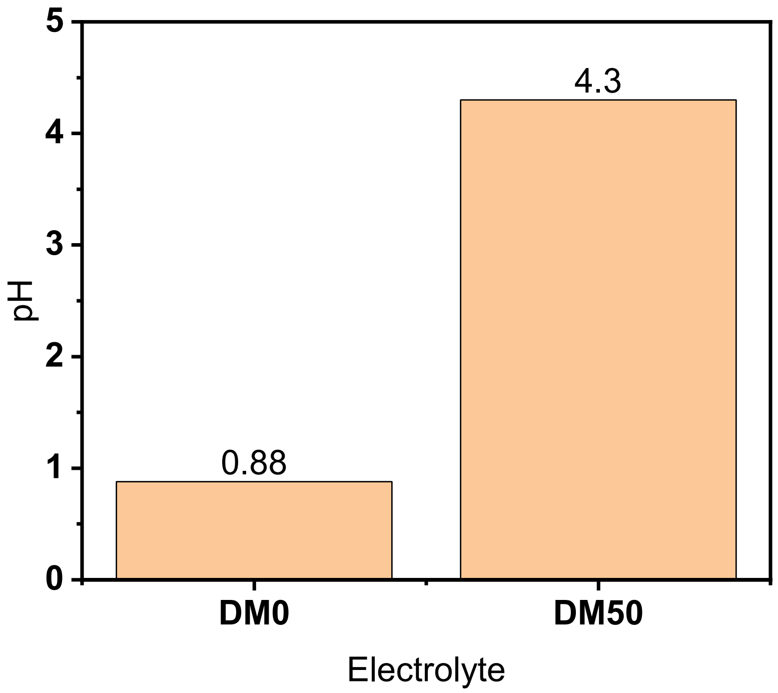

Figure S3. pH measurement for different electrolytes (measured at 20°C).

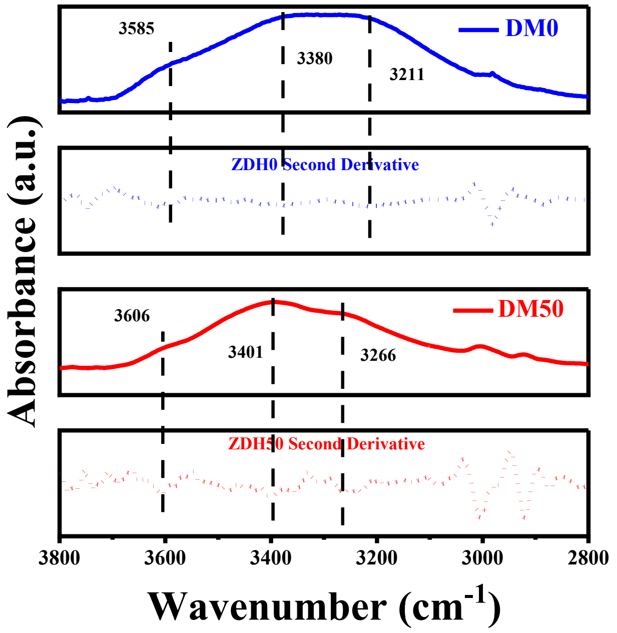

Figure S4. Secondary derivative of FTIR curves showing the states of different hydrogen bonding.

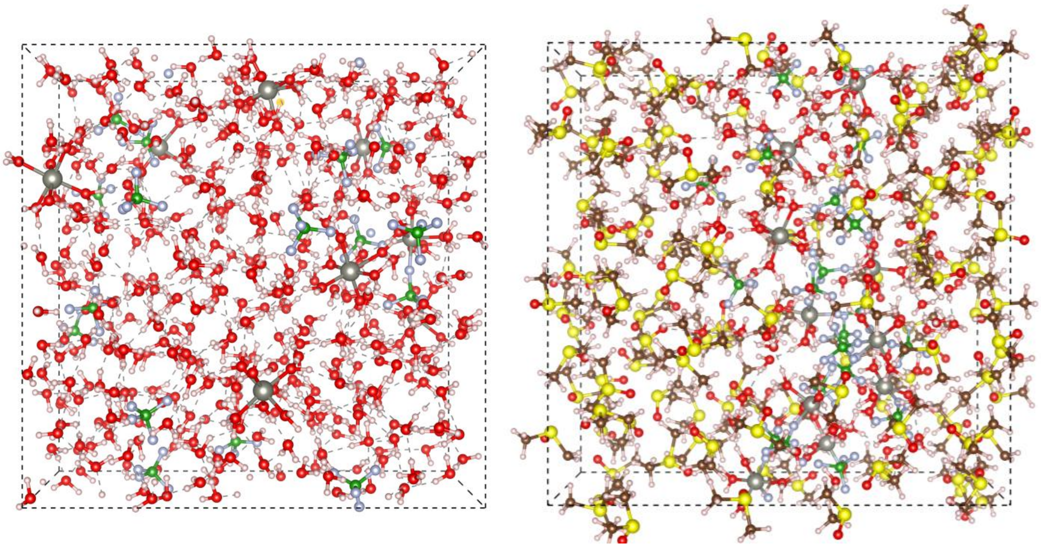

Figure S5. MD simulation cells for $Zn(BF_4)_2/H_2O$ (left) and $Zn(BF_4)_2/H_2O/DMSO$ (right).

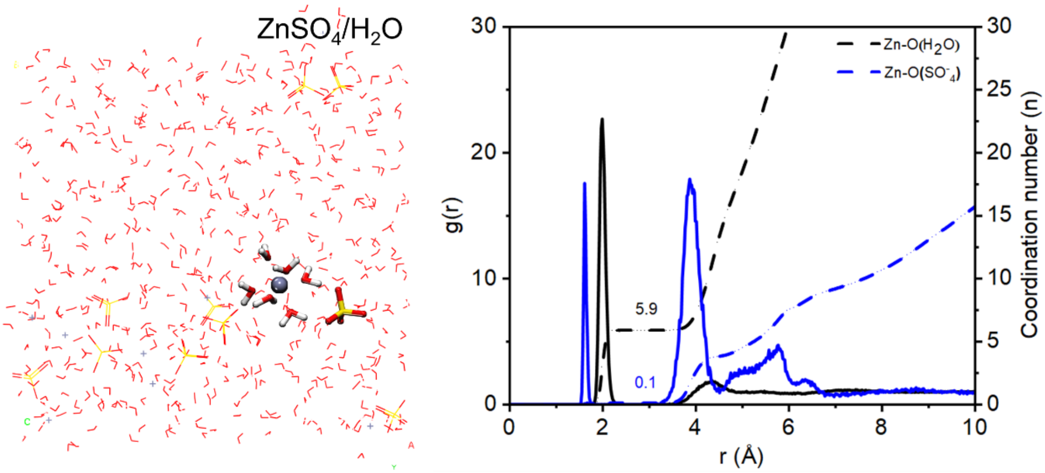

Figure S6. MD simulation, CN, and RDF for $ZnSO_4/H_2O$.

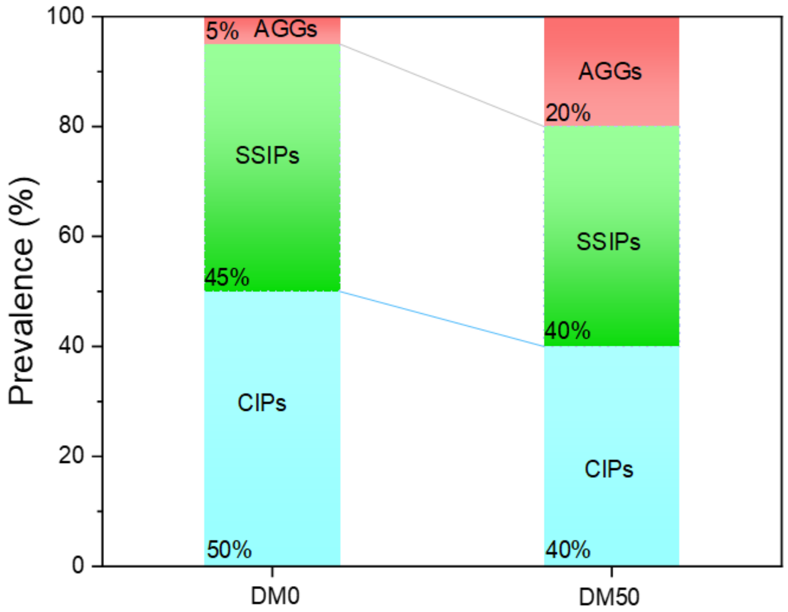

Figure S7. The solvation structure analysis of DM0 and DM50 electrolytes. The population of SSIP, CIP, and AGG species in two types of electrolytes.

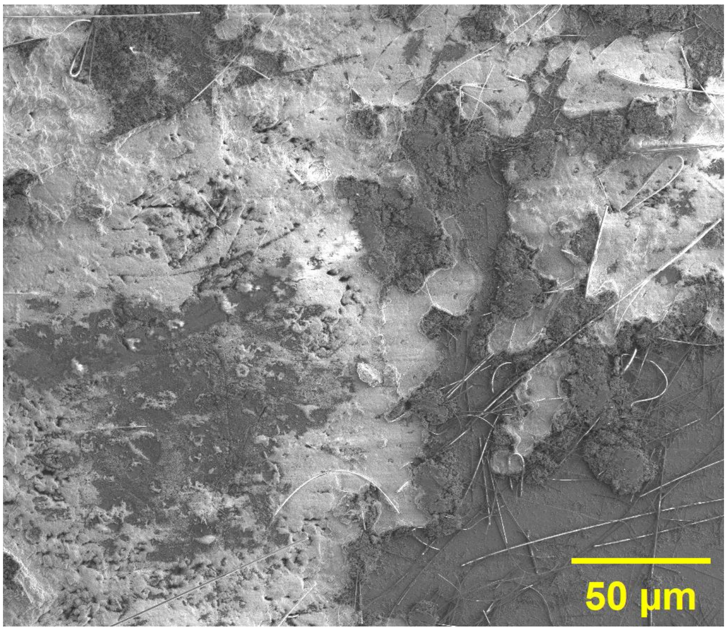

Figure S8. SEM images for Zn anode after 5 cycles at 3 mA cm^{-2} and 3 mAh cm^{-2} in 0.5M $Zn(BF_4)_2$ in H_2O (aqueous ZBF)

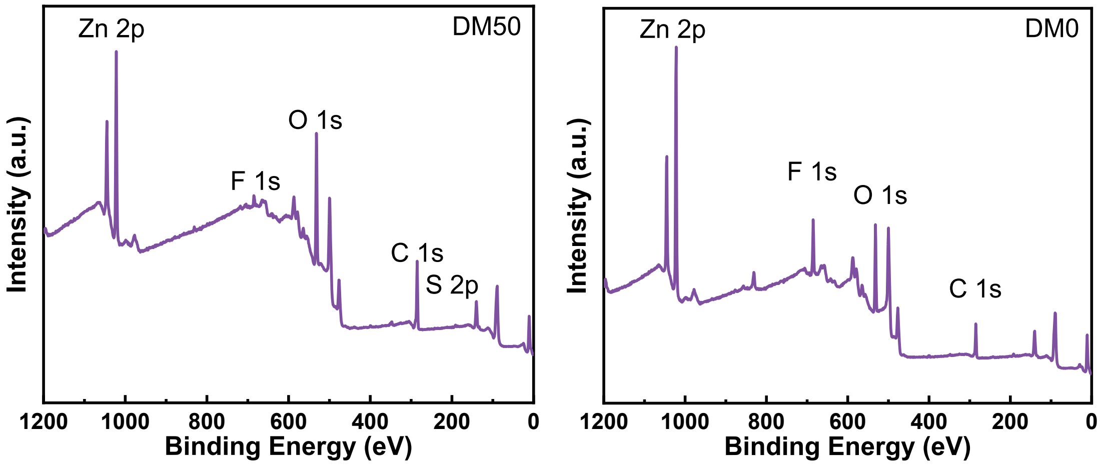

Figure S9. XPS survey for DM50 (left) and DM0 (right).

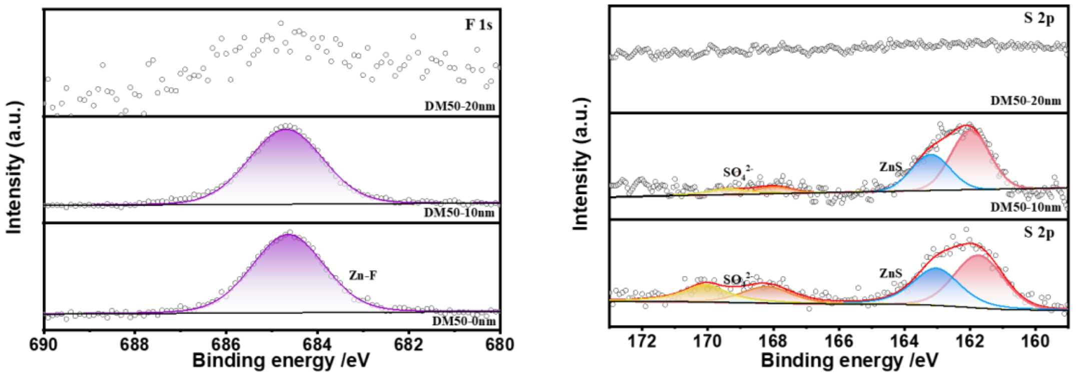

Figure S10. XPS depth profiling of the Zn anode cycled in the DM50 electrolyte. Spectra of F 1s (left) and S 2p (right) regions at different Ar^+ sputtering depths.

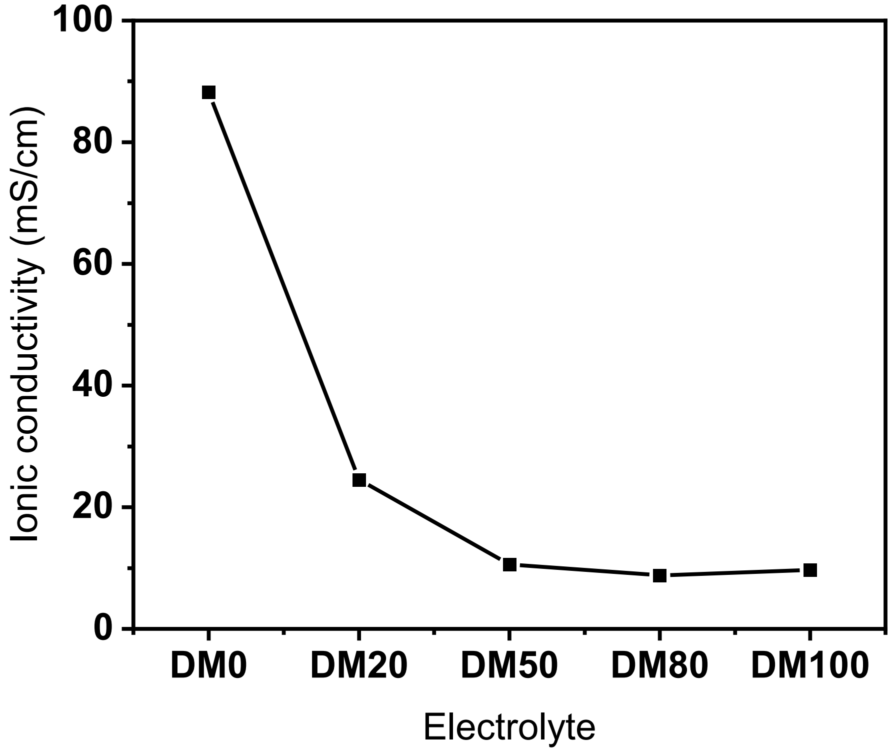

Figure S11. Ionic conductivity of different electrolytes (measured at 23°C)

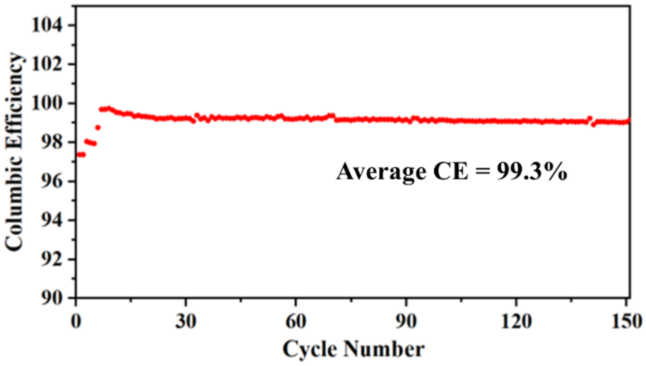

Figure S12. Columbic efficiency of DM50, tested in Zn//Cu asymmetric cells at the current density of 3 mA cm^{-2}

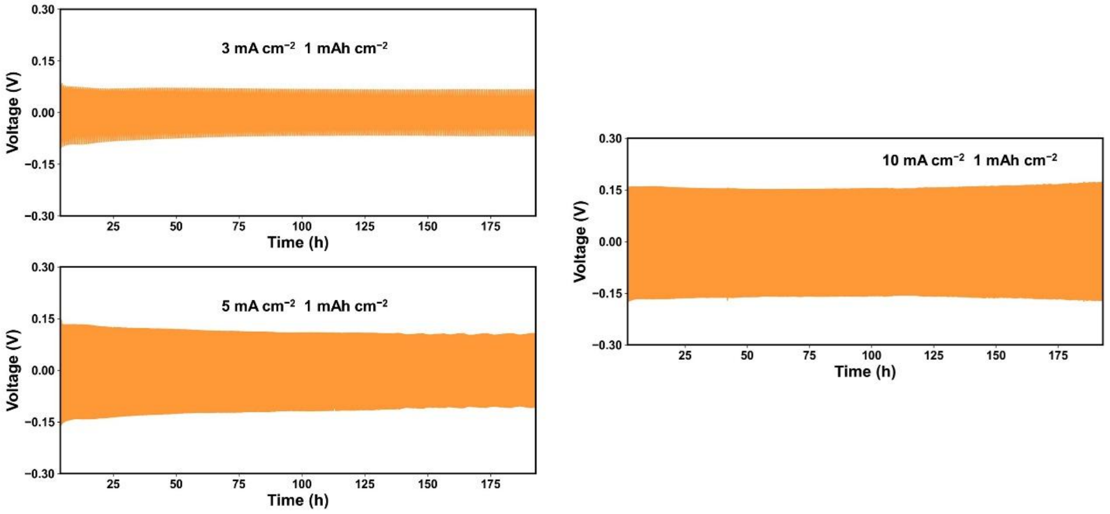

Figure S13. Galvanostatic cycling profiles of Zn||Zn symmetric cells in the DM50 electrolyte at current densities of 3 mA cm^{-2}, 5 mA cm^{-2}, and 10 mA cm^{-2} with a fixed areal capacity of 1 mAh cm^{-2}. All cells exhibit stable and low-polarization voltage profiles.

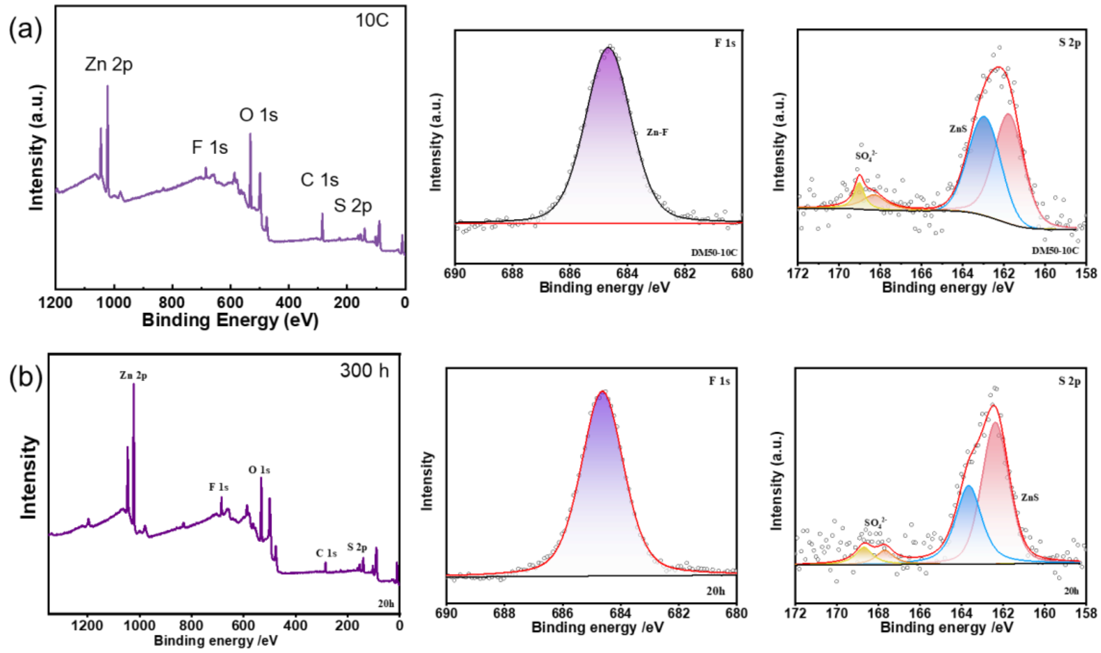

Figure S14. Long-term cycling and high-rate stability of SEI: (a) (Left) XPS spectra of the Zn anode after cycling at 10C condition for 10 hours in the DM50 electrolyte. (Right) XPS spectra of F 1s and S 2p regions, respectively. (b) (Left) XPS spectra of the Zn anode after cycling at 1C condition for 300 hours in the DM50 electrolyte. (Right) XPS spectra of F 1s and S 2p regions, respectively.

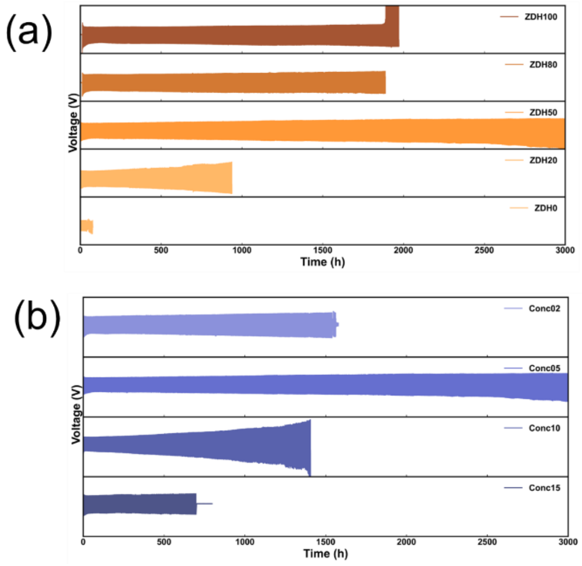

Figure S15. Symmetric cells cycled at 3 mA cm^{-2} and 3 mAh cm^{-2} with different (a) solvent ratios and (b) $Zn(BF_4)_2$ concentrations.

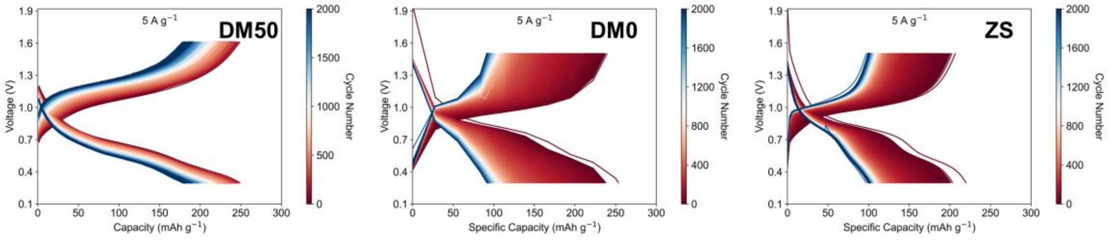

Figure S16. Charge-discharge curves of the Zn||NVO cell with different electrolytes at a current density of 5 A g^{-1}.

Supplementary Tables

Table S1. Market price of current used salts in ZIBs

Solute	$Zn(OAc)_2$	*$Zn(BF_4)_2$	$ZnSO_4$	$ZnCl_4$	$Zn(CF_3SO_3)_2$	*$Zn(ClO_4)_2$	*$Zn(TFSI)_2$
¥/kg	143.8	419.9	734.8	1540.9	2639.8	6400	32000
$/kg	20.11189	58.73	102.7692	215.5105	369.2028	895.1049	4475.524

Salt prices (with the same package size) were obtained from two major suppliers in China, Aladdin (https://www.aladdin-e.com/) and Aicaigou (https://b2b.baidu.com/). Salts marked with an asterisk (*) were obtained from Aicaigou.

Table S2. Radar Chart Scoring Criteria

Aspect	5	3-4	1-2	Conventional Aqueous System	Organic System	Other System containing $Zn(BF_4)_2$	This Work
**Cost**	Lowest cost (significantly lower than other systems)	Moderate cost (comparable to other competitive systems)	High cost (uses expensive materials, limiting commercial viability)	5	1	4	4
**Safety**	Excellent safety (non-flammable, no toxic byproducts, stable)	Moderate safety (some risk of flammability or toxicity)	Poor safety (high flammability, hazardous to handle or dispose of)	5	2	5	5
**Cycling Performance**	High accumulative capacity, stable cycling	Moderate accumulative capacity with reasonable	Low accumulative capacity with performance	2	5	2	4

	performance at practical conditions	cycling stability	prone to degradation at high current				
**Stable SEI**	Stable SEI formation (dense, compact SEI that remains intact during cycling)	Thick or partially unstable SEI formation	No SEI layer or porous SEI formed during cycling	1	5	5	5
**Cathode Compatibility**	Long cycling life with high capacity retention and minimal cathode dissolution	Moderate compatibility with some capacity fade or minor dissolution	Poor compatibility, significant capacity fade, or rapid cathode dissolution	2	4	5	5
**Side Reaction Resistance**	High resistance to side reactions (e.g., HER, corrosion suppression)	Moderate resistance to side reactions with manageable degradation	Low resistance, significant occurrence of side reactions (e.g., HER, corrosion)	2	5	5	5

As shown in **Table S1 and S2**, the common aqueous electrolyte, being the earliest and most widely used system, offers advantages in terms of low cost and high safety. However, it suffers from significant performance drawbacks due to issues such as cathode dissolution and severe side reactions.

The common organic electrolyte system typically forms a stable SEI, effectively mitigating the issues faced by aqueous electrolytes and providing improved cycling performance. However, the organic solvents used are generally flammable, and the compatible organic salts are often expensive, which conflicts with the intention of using ZIBs as a low-cost alternative to LIBs for large-scale energy storage applications.

The introduction of $Zn(BF_4)_2$ addresses the high cost of organic salts, and as $Zn(BF_4)_2$ is commonly used as a flame retardant, it significantly enhances the safety of the electrolyte system. However, a major challenge remains due to the lack of compatible solvents, which means that the performance still lags behind that of current organic electrolyte systems.

In contrast, this work presents an effective solution by introducing a DMSO/H_2O solvent system, which retains the cost and safety advantages of ZBF while significantly enhancing performance. The combination of DMSO and H_2O not only stabilizes the solvation structure but also facilitates the formation of a robust SEI, thereby improving the overall cycling stability and efficiency of the electrolyte system.

Table S3. References used for the radar chart

Reference	Electrolyte	Accumulative capacity	SEI	Safety
This work	Zn(BF4)2 / DMSO	4500	Compact SEI	Non-flammable
Conventional Aqueous electrolyte	ZnSO4 / H2O	330	Porous Surface	Non-flammable
Ref[2]	Zn(OTf)2 / DMF	2700	Compact SEI	Non-flammable
Ref[3]	Zn(OTf)2 / DME	5000	Stable SEI	Flammable
Ref[4]	Zn(OTf)2 / PC	2000	Stable SEI, Hydrophobic	Flammable
Ref[5]	Zn(TFSI)2 / DMSO	1000	Compact SEI	Non-flammable
Ref[6]	Zn(BF4)2 / EG	1000	Compact, dense ZnF_2-based	Non-flammable
Ref[7]	Zn(BF4)2 / GBL	2500	Dense, ZnF_2-rich	Low flammability
Ref[8]	Zn(BF4)2 / VC	550	Dense, ZnF_2/$ZnCO_3$ hybrid SEI	Low flammability
Ref[9]	Zn(BF4)2 / DMC / EC	720	Stable, Zn^{2+}-EC/DMC pairing	Flammable
Ref[10]	Zn(BF4)2 / THF	312	Dense, compact ZnF2-based SEI	Low flammability

References

1. Shang, Y. & Kundu, D. A path forward for the translational development of aqueous zinc-ion batteries. *Joule* **7**, 244–250 (2023).
2. Wang, N. *et al.* Zinc–Organic Battery with a Wide Operation-Temperature Window from −70 to 150 °C. *Angew. Chem. Int. Ed.* **59**, 14577–14583 (2020).
3. Ma, G. *et al.* Reshaping the electrolyte structure and interface chemistry for stable aqueous zinc batteries. *Energy Storage Mater.* **47**, 203–210 (2022).
4. Ming, F. *et al.* Co-Solvent Electrolyte Engineering for Stable Anode-Free Zinc Metal Batteries. *J. Am. Chem. Soc.* **144**, 7160–7170 (2022).
5. Jian, Q., Wang, T., Sun, J., Wu, M. & Zhao, T. In-situ construction of fluorinated solid-electrolyte interphase for highly reversible zinc anodes. *Energy Storage Mater.* **53**, 559–568 (2022).
6. Han, D. *et al.* A non-flammable hydrous organic electrolyte for sustainable zinc batteries. *Nat. Sustain.* **5**, 205–213 (2021).
7. Yang, W. *et al.* Synergistic Cation Solvation Reorganization and Fluorinated Interphase for High Reversibility and Utilization of Zinc Metal Anode. *ACS Nano* **17**, 25335–25347 (2023).
8. Wang, S. *et al.* Highly reversible zinc metal anode enabled by zinc fluoroborate salt-based hydrous organic electrolyte. *Energy Storage Mater.* **63**, 102971 (2023).
9. He, R. *et al.* A Dual Organic Solvent Zn-Ion Electrolyte Enables Highly Stable Zn Metal Batteries. *Nano Lett.* acs.nanolett.3c01406 (2023) doi:10.1021/acs.nanolett.3c01406.

10. Li, D. *et al.* Regulating Zn^{2+} Solvation Shell Through Charge-Concentrated Anions for High Zn Plating/Stripping Coulombic Efficiency. *Adv. Funct. Mater.* **34**, 2405145 (2024).
